# Calpain-5 gene expression in the mouse eye and brain

**DOI:** 10.1186/s13104-017-2927-8

**Published:** 2017-11-21

**Authors:** Kellie Schaefer, MaryAnn Mahajan, Anuradha Gore, Stephen H. Tsang, Alexander G. Bassuk, Vinit B. Mahajan

**Affiliations:** 1 Omics Laboratory, Department of Ophthalmology, Byers Eye Institute, Stanford University, Palo Alto, CA 94304 USA; 2 Palo Alto Veterans Administration, Palo Alto, CA USA; 3 Bernard and Shirlee Brown Glaucoma Laboratory, Department of Pathology and Cell Biology, Department of Ophthalmology, College of Physicians and Surgeons, Columbia University, New York, NY USA; 4 Department of Pediatrics, University of Iowa, Iowa City, IA USA

**Keywords:** CAPN5, Calpain, In situ hybridization, Retina, Brain, Gene expression

## Abstract

**Objective:**

Our objective was to characterize *CAPN5* gene expression in the mouse central nervous system. Mouse brain and eye sections were probed with two high-affinity RNA oligonucleotide analogs designed to bind *CAPN5* RNA and one scramble, control oligonucleotide. Images were captured in brightfield.

**Results:**

*CAPN5* RNA probes were validated on mouse breast cancer tumor tissue. In the eye, *CAPN5* was expressed in the ganglion cell, inner nuclear and outer nuclear layers of the retina. Signal could not be detected in the ciliary body or the iris because of the high density of melanin. In the brain, *CAPN5* was expressed in the granule cell layers of the hippocampus and cerebellum. There was scattered expression in pons. The visual cortex showed faint signal. Most signal in the brain was in a punctate pattern.

## Introduction

Calpain-5 (CAPN5) is a member of the calpain family of calcium-activated proteases that target a variety of pathways to exert control over numerous processes, including tissue necrosis, cytoskeletal remodeling, cell-cycle control, cell migration, myofibril turnover, regulation of gene expression, apoptosis, long-term potentiation, and signal transduction [1]. Many eye-related pathologies are associated with increased calpain activity. These include retinal hypoxia [2–5], retinal degeneration [6, 7], retinal detachment [8], retinitis pigmentosa [9–11], and glaucoma [12, 13]. Increased calpain activity is also associated with neurological diseases including, Huntington’s disease [14], Alzheimer’s disease [15, 16], multiple sclerosis [17], and traumatic brain injury [18, 19]. Autosomal dominant neovascular inflammatory vitreoretinopathy (ADNIV) is caused by single point mutations in the *CAPN5* gene (OMIM #602537) [20–22], which causes the CAPN5 protease to become hyperactive. ADNIV patients experience sequential uveitis, retinitis pigmentosa, retinal neovascularization, and proliferative retinopathy. Which ultimately leads to blindness [20]. Currently there is no treatment.

An important question to understanding how CAPN5 leads to disease is identifying which tissues CAPN5 is expressed in and the levels of CAPN5 in those tissues. Previous studies have used RT-PCR to detect relative levels of *CAPN5* in human and rat brains, with results showing a wide expression profile in rat and human brains [23, 24]. Others have used immunohistochemistry (IHC) to detect CAPN5 in the retina of mice, which showed expression varied based on the antibody used [20, 22, 25]. Although these were important initial studies, a more complete picture is needed to understand CAPN5 expression in the central nervous system. In situ hybridization (ISH) offers some advantages over RT-PCR and IHC. ISH allows for detecting mRNA levels in an intact tissue, something RT-PCR does not. This is more specific because it produces an image that differentiates between specific cell types within a tissue and even cellular compartments within a cell type. ISH can also complement IHC data by detecting mRNA expression whereas IHC detects protein expression. Additionally, while multiple antibodies are available for CAPN5, they have previously been shown to give different expression patterns [22, 25]. For this reason, mRNA in situ hybridization experiments were performed to identify the expression of *CAPN5* in the mouse eye and brain.

## Main text

### Methods

#### Animal Care and euthanasia

Ten-week-old C57BL/6 mice were procured from the Toronto Centre for Phenogenomics. Mice were housed in a standard 12 h light/dark cycle. Healthy mice were euthanized by lethal intraperitoneal injection of sodium pentobarbital. Once animals were deeply anesthetized, the thoracic cavity was opened by a ventral midline incision and a small cut in the right atrium was made for blood outflow. 10 mL of PBS was perfused by a 25 g needle through the left ventricle. Then perfusion of 10% buffered formalin was performed. Organs were harvested and fixed overnight at 4 °C.

#### Tissue description and treatment

Tissue sections were cut on a Microm HM 355S microtome (ThermoFisher, Waltham, MA) to a thickness of 10 microns. Formalin-fixed paraffin-embedded sections from C57Bl/6 mice were deparaffinized for 5 min in xylene, immersed in 100% ethanol for 5 min then air-dried. Treatment was with Bond Epitope Retrieval Solution 2 (AR9640, Leica Biosystems, Buffalo Grove, IL) for 30 min.

#### In situ hybridization with LNA probes

High-affinity RNA oligonucleotide analogs (Locked Nucleic Acid, LNA™, Exiquon, Denmark) were designed to bind CAPN5 RNA. The proprietary Exiquon LNA™ probe designer software was used to design custom probes to target *CAPN5,* while limiting non-specific binding. A probe cocktail of calpain-5 probe-1 (*5DigN/TGATACACAGCGGAAGTGGT)* and calpain-5 probe-2 (*5DigN/ACCAGAGGCAGAGTGTAACAGT*) (probe cocktail), and a scramble-miR (*5DigN/GTGTAACACGTCTATACGCCCA*), were prepared according to the manufacturer’s recommended conditions (Exiqon, Denmark), and each was labeled at the 5′ end with digoxigenin [26]. All experimental tissue sections were probed with a cocktail of both probes 1 and 2.

#### Hybridization and washing procedures

Probes were resuspended in 10 μl then diluted 1:25 in Enzo hybridization buffer (ENZ-33808, Enzo Life Sciences, Farmingdale, NY), placed on tissue sections, covered with polypropylene coverslips and heated to 60 °C for 5 min, followed by hybridization at 37 °C overnight. Sections were washed in intermediate stringency solution (0.2× SSC with 2% bovine serum albumin) at 55 °C for 10 min.

#### Immunohistochemistry and color development

Sections were treated with anti-digoxigenin–alkaline phosphatase conjugate (1:150 dilution in pH 7 Tris buffer; Roche, Switzerland) at 37 °C for 30 min. Development was carried out with NBT/BCIP (34042, ThermoFisher, Waltham, MA), closely monitored and stopped when the control sections appeared light blue. Development time with the chromogen was between 15 and 30 min. Sections were counterstained with nuclear fast red (N3020, Sigma-Aldrich, St. Louis, MO) for 3–5 min, rinsed and mounted with coverslips.

#### Imaging

**Fig. 1:**
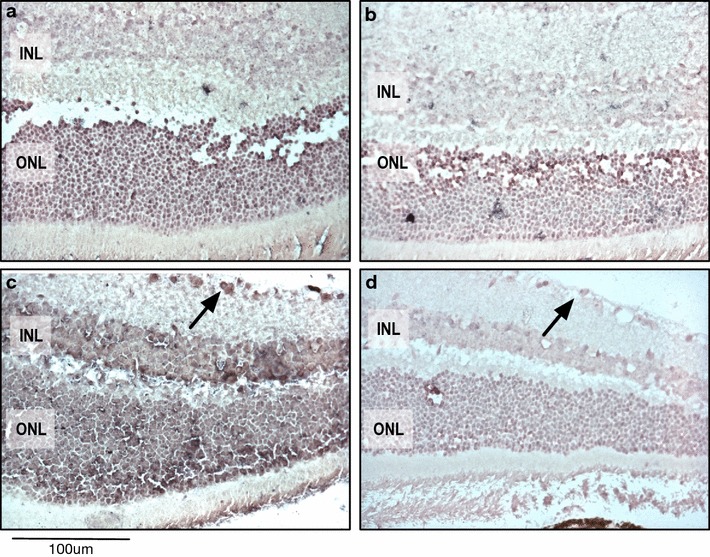
CAPN5 mRNA expression in the retina. **a** Calpain 5 signal was detected in the inner nuclear layer (INL) and outer nuclear layer (ONL) of the central retina. **b** Scramble-miR (control) in a section adjacent to **a**. **c** Peripheral retina highlights *CAPN5* signal in ganglion cell layer (arrow). **d** Scramble-miR (control) in a section adjacent to **c**

**Fig. 2:**
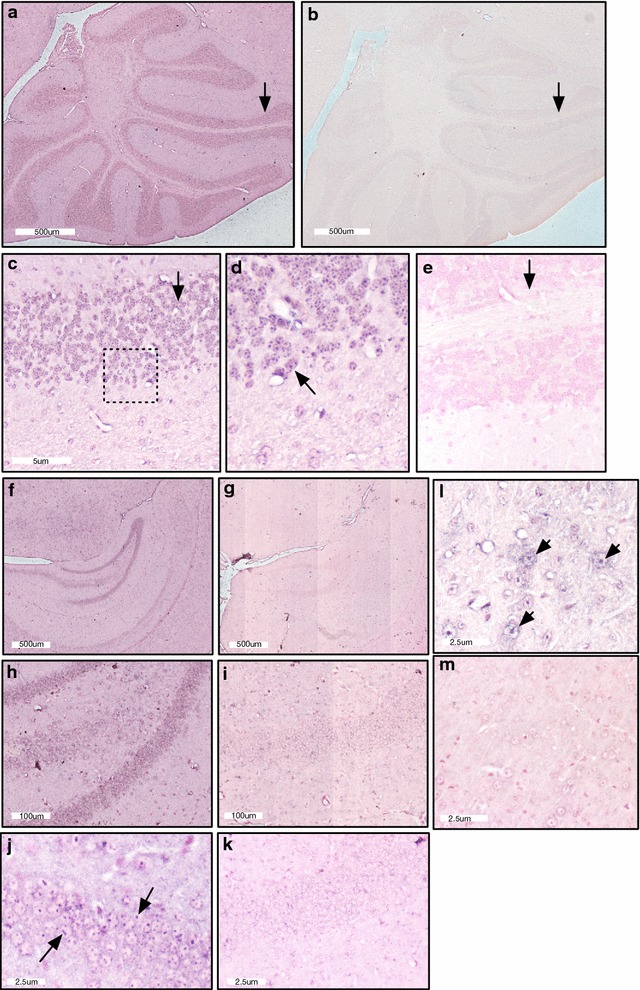
CAPN5 mRNA expression in the brain. **a**–**e** CAPN5 mRNA expression in the cerebellum. **a** *CAPN5* signal is concentrated in the granule cell layer (arrows) in the cerebellum. **b** Scramble-miR (control) in a section adjacent to **a**. **c** Higher magnification of **a**. **d** Higher magnification of **c**. Note the punctate signal (arrow). **e** Scramble-miR (control) corresponding to **c**. **f**–**k** CAPN5 mRNA expression in the hippocampus. **f** *CAPN5* signal is concentrated in the granule cell layer in the hippocampus. **g** Scramble-miR (control) in a section adjacent to **f**. **h** Higher magnification of **f**. **i** Scramble-miR (control) corresponding to **h**. **j** Calpain 5 signal seen in the granule cell layer at high magnification. Note the punctate signal (arrows). **k** Scramble-miR (control) in a section adjacent to **j**. **l** and **m** CAPN5 mRNA expression in the pons. **l** *CAPN5* signal seen in larger neurons at high magnification. Note the punctate signal (arrows). **m** Scramble-miR (control) in a section adjacent to **l**

Brightfield images for Figs. 1 and 2a, b, f–i were extracted from Leica ScanScope XT slide scans (Leica Biosystems, Buffalo Grove, IL) or on Zeiss Axio A1 (Zeiss Microscopy, Germany). Images for Fig. 2c–e, j–m were taken on a brightfield microscope (Zeiss Microscopy, Germany). All images were saved in jpeg format.

### Results

**Fig. 3:**
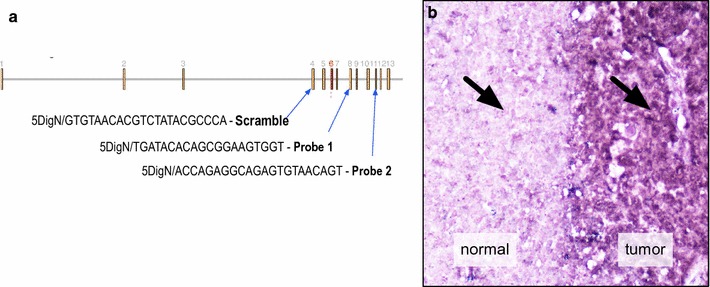
Probes used for in situ assays. **a** The *CAPN5* gene contains 13 exons (brown). The ADNIV associated mutations occur in exon 6 (red). The three probes correspond to three different spots within the *CAPN5* gene. The control scramble probe corresponds to the scrambled version of a sequence in exon 4. Probe 1 corresponds to the indicated sequence in exon 8, and Probe 2 corresponds to the indicated sequence in exon 11. **b** The probes were tested by application to normal tissue (left) and breast cancer tumor tissue (right)

To better understand *CAPN5* expression in the central nervous system, an in situ hybridization assay was developed. Two *CAPN5* oligonucleotide probes were designed along with a negative control scramble oligonucleotide probe (Fig. 3a). To validate the assay, a cocktail of *CAPN5* probes 1 and 2 were first applied to mouse breast cancer sections (determined histologically from a tumor), since calpain expression is linked to a variety of cancers [27–29]. *CAPN5* expression was detected in the cancerous tissue but not the normal tissue (Fig. 3b). No signal was observed using the scramble probe (data not shown).

Next, we studied *CAPN5* expression in the retina (Fig. 1). Previously, we reported that protein expression detection varied depending on which antibody was used [20, 22, 25]. *CAPN5* mRNA signal was detected in the ganglion cell, inner nuclear and outer nuclear layers of the retina. This corroborated with our most recent IHC data indicating that CAPN5 is expressed in all layers of the retina, even though the phenotype is largely restricted to the photoreceptors [25]. Signal was not detected in the lens or cornea (data not shown). Because of the high density of melanin in the ciliary body, iris, and RPE, the in situ probes signal could not be ascertained, but previous IHC studies did not identify CAPN5 protein in these structures.

Punctate signal was seen in the hippocampus and cerebellum. In each of these sites, signal was localized to the granule cell layers. Specifically, *CAPN5* signal was detected in CA3 and the dentate gyrus of the hippocampus. Scattered large neurons in pons also showed punctate signal. There appeared to be some faint signal in the visual cortex, and there was no signal detected in the auditory cortex, hypothalamus or striatum (data not shown).

### Discussion

Identifying target cells and cellular localization is important for gaining better insights into protein function when considering therapeutic intervention. Previous studies have reported a number of tissue types and cell types in the central nervous system to contain CAPN5 depending on the method used to detect it. RT-PCR showed *CAPN5* is present at relatively high levels in the rat and human brain. It is the second highest expressed calpain in the rat brain, following *CAPN2.* Additionally, *CAPN5* mRNA was detected ubiquitously in rat brain, but was only found in the frontal lobe, cerebellum, medulla, hypothalamus and thalamus in human brain. IHC studies revealed CAPN5 in the inner and outer segments (IS and OS), the inner and outer plexiform layers (IPL and OPL), inner nuclear layer (INL) and retinal ganglion cells (RGC), depending on the antibody used [20, 22, 25].

Studies in the EMBL-EBI gene expression database report relative tissue expression data collected from RNA-seq and microarray experiments [30, 31]. In mice, twelve studies investigating *CAPN5* tissue expression were found on the EMBL-EBI database. In contrast to other data, these studies found a stark predominance of *CAPN5* expression in brain tissue. The EMBL-EBI database included eight experimental datasets for human *CAPN5* expression. Brain *CAPN5* expression was also found in human datasets on EMBL-EBI, though at levels much more modest than in mice relative to other tissues investigated.

The results obtained in the current study are in agreement to previous IHC experiments. Both methods detected *CAPN5* in retinal ganglion cells and the INL. However, our study also detected *CAPN5* in the ONL, but not the IS and OS or the IPL and OPL. In agreement with RT-PCR experiments, *CAPN5* was detected in the cerebellum, specifically the granular cell layer. We also detected expression in the hippocampus and pons, but did not detect significant expression in any other regions.

Our results support our previous finding that CAPN5 may be playing a role in phototransduction. *CAPN5* was detected in the INL, ONL and retinal ganglion cells. The INL is made up of the cell bodies of horizontal cells, amacrine cells and bipolar cells. The ONL contains the cell bodies of the two types of photoreceptors, rods and cones. Although we previously reported CAPN5 at the photoreceptor synapses, because in situ detects the mRNA of the protein, it’s possible once *CAPN5* is translated in the cell bodies, it is transported to the synapses. This hypothesis could also be true for *CAPN5* expression in retinal ganglion cells, the cell type which forms the optic nerve.

The role of CAPN5 in the brain is less clear. The pons serves as a bridge between the cerebellum and forebrain. It controls many basic functions of the body including the basic senses, sleep, respiration and posture. The hippocampus is involved in memory, specifically long-term memory, while the cerebellum regulates motor movements, balance and speech. One hypothesis, based off the apparent role of CAPN5 in the eye, is that CAPN5 may play a role in signal transduction in the brain as well. This is supported by the fact that *CAPN5* is seen at incredibly high levels in granule cells of the cerebellum, the most numerous type of neurons. Additionally, granule cells are believed to fine-tune inputs from the brain by allowing for combinatorial coding [32]. Further support for this hypothesis is the presence of *CAPN*5 in the dentate gyrus and CA3 subfield, which is the most interconnected region of the hippocampus. Nonetheless, patients with CAPN5 mutations to date do not display brain-related phenotypes, suggesting the effect of hyperactive CAPN5 is nondetrimental in the brain.

Although this study reported *CAPN5* expression in the cerebellum, pons and hippocampus, ADNIV patient phenotypes are restricted to the eye. There may be excess calpain activity in the retina, due to the high level of calcium required for phototransduction; and the brain may be more resistant to CAPN5 damage since there are comparatively fewer cells in the granule cell layer expressing *CAPN5*. Another possibility is that CAPN5 has different substrates in the brain and eye, and one or more of the eye substrates may have a more detrimental effect when misregulated by a hyperactive CAPN5. Additional studies will need to be performed to determine the substrates of CAPN5.

Although some studies suggest ISH may give similar results to IHC, it seems to depend on the antibody used and is mainly analyzed in a present-or-not manner rather than in a qualitative manner [33–35]. Additionally, it is important to note that there may be differences in mRNA expression versus protein expression depending on the tissue. Therefore, in analyzing expression throughout a whole tissue, ISH in conjunction with IHC may give the most complete picture.

### Conclusions

In the eye, *CAPN5* mRNA was seen in ganglion cell, inner nuclear and outer nuclear layers of the retina. In the brain, signal was evident in the granule layers of the cerebellum and hippocampus and in scattered large neurons in pons. Our findings support the concept that CAPN5 may be playing a role in signal transduction in both the brain and eye. With this expression data in mind, future studies may begin to narrow down potential substrates of CAPN5, and better understand the underlying mechanism of its pathology. Ultimately, a better understanding of CAPN5 may provide useful drug targets as treatments for the many pathologies related to CAPN5.

## Limitations

This study is limited to expression of *CAPN5* in mice. There may be minor differences in human expression. This data and its relationship to CAPN5-associated diseases may be further interpreted once the substrates of CAPN5 have been identified. Future studies can examine other calpains in regions of the brain including a more detailed look at the cerebral cortex.

## Abbreviations

CAPN5: calpain 5
IHC: immunohistochemistry
ISH: in situ hybridization
ADNIV: autosomal dominant neovascular inflammatory vitreoretinopathy
IS: inner segment
OS: outer segment
IPL: inner plexiform layer
OPL: outer plexiform layer
INL: inner nuclear layer
RGC: retinal ganglion cells
